# Direct Imaging and Location of Pb^2+^ and K^+^ in EMT Framework-Type Zeolite

**DOI:** 10.1021/acs.jpcc.1c00550

**Published:** 2021-03-15

**Authors:** Yaping Zhang, Daniel Smith, Jennifer E. Readman, Alvaro Mayoral

**Affiliations:** † Instituto de Nanociencia y Materiales de Aragón (INMA), CSIC-Universidad de Zaragoza, Zaragoza 50009, Spain; ‡ Center for High-Resolution Electron Microscopy (CℏEM), School of Physical Science and Technology (SPST), ShanghaiTech University, 393 Middle Huaxia Road, Pudong, Shanghai 201210, China; § Advanced Microscopy Laboratory (LMA), University of Zaragoza, Zaragoza 50018, Spain; ∥ School of Physical Sciences and Computing, University of Central Lancashire, Preston PR1 2HE, United Kingdom

## Abstract

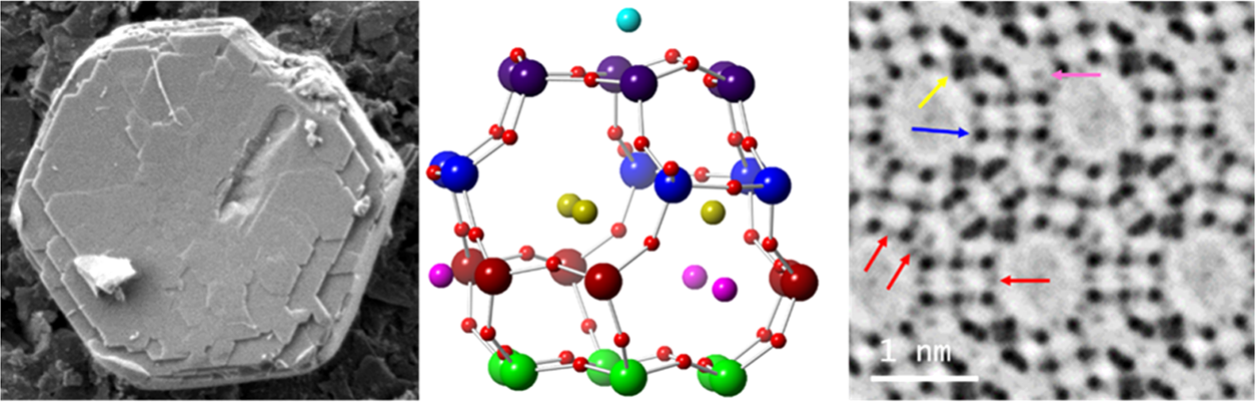

The understanding of the structural framework and metal locations within zeolites are two critical aspects to tune and further exploit their properties as heterogeneous catalysts, advanced optical devices, or as water remediation materials. The development of electron microscopy has made it possible to observe directly the zeolitic framework and the distribution of extra framework cations within the pores at the atomic level. Here, we have studied the **EMT** framework providing data with an unprecedented spatial resolution, which have allowed the analysis of the **FAU** domains present in the framework. Additionally, potassium and lead (introduced by aqueous ion exchange), which were nonperiodically distributed in the pores have been located. An alternative image mode, annular bright field, has been used, proving its usefulness to extend the spatial resolution and increase the sensitivity toward light elements such as bridging oxygen. Finally, the combination of atomic imaging (local information) with the three-dimensional electron diffraction tomography analysis (averaged information) determined the lead-**EMT** crystal symmetry to be **P**6_3_**mc**.

## 1 Introduction

Zeolites are microporous materials with tunable porosity widely used as ion exchangers or heterogeneous catalysts^1^ among many other applications. They are able to confine elements within their pores and also exhibit very good thermal stability.^2−4^ Due to the confinement capability that zeolites exhibit, they can protect extra framework species, minimizing their agglomeration, and release back to the environment as well as tuning their physical properties (for example, work function or luminescence).^5−18^

There are currently up to 253 different zeolite topologies^19^ in the International Zeolite Association (IZA) structure database; among them, **TSC**, **EMT**, and **FAU** exhibit the lowest framework densities of 13.2–13.3 T/1000 Å^3^ of the aluminosilicate family. Lower density zeolites are highly desirable for soil treatment applications^20^ or catalysis.^21^**EMT** was first synthesized in 1971^22^ as a mixture of **FAU** and **EMT**. The pure **EMT** framework, named EMC-2 after **E**lf (or Ecole Supérieure) **M**ulhouse **C**himie-two, was obtained by Delprato and co-workers^23^ in 1990 and the structure was completely solved by Baerlocher et al. 4 years later.^24^**EMT** is the hexagonal polytype of the **FAU**-type zeolite (cubic) sharing the same layer units composed of sodalite cages (*t-toc*) linked by double six-membered rings (d6R, *t-hpr*). The **EMT** framework contains large *t-wof* and *t-wou* cages with three and five twelve-membered ring (12MR) windows, respectively. In contrast with the zigzag channels of **FAU** and the eight-membered ring windows of **TSC**, **EMT** has straight access along three directions, all with 12MR windows. **EMT** has previously been used in biomedicine^25^ and gas capture and separation.^26^ It also shows great potential for industrial applications such as petroleum cracking due to its large channel and cavity size.

Besides the use of zeolites as heterogeneous catalysts and water softeners, the capability of zeolites to capture different elements within their pore system is also attractive for various applications such as optoelectronics or water remediation. The interest of occluding lead within the pores of zeolites or metal–organic frameworks (MOFs) has regained much interest due to the possibility of tuning the optical response of lead-loaded zeolites while preventing lead discharge into the environment due to its coordination to the framework. The incorporation of lead species can be summarized in three types: (i) unitary: clusters (including single atom) with only one element;^14,27^ (ii) binary: species formed by two elements, such as PbS and PbI_2_;^28−31^ and (iii) ternary: lead compounds formed by three elements, CsPbI_3_ and CsPbBr_3_.^32^

To gain structural information, there are several characterization methods available to help explain the physicochemical properties of zeolites. Such properties are strongly affected by the type of metal introduced, its size, and location; therefore, characterization at the atomic level is becoming indispensable to tailor the properties of materials at the nanoscale. Zeolite frameworks present high degree of symmetry, whereas the exchanged forms are usually disordered. Therefore, long-range techniques such as X-ray diffraction and Rietveld analysis do not always give the short-range information needed. In this sense, transmission electron microscopy is the most powerful methodology as it can provide direct visualization of the framework and the guest species at an atomic level together with crystallographic and chemical information.^33−37^ All of these results have been achieved with thorough control of the electron dose, as zeolites are strongly beam sensitive. Working in the probe mode offers a quick scan, reducing the sample damage. The possibility of obtaining sub-Ängstrom probes together with the use of several detectors simultaneously allows for the collection of signals at a high angle (annular dark field, ADF), providing information regarding the heavy elements. Meanwhile, an annular bright-field detector (ABF) enhances the signal of light compounds,^38,39^ which are scattered at low angle. Therefore, by combining the information from both detectors, determining the location and element type of both light and heavy elements is achievable.

In this work, **EMT** framework-type zeolite was ion exchanged with Pb(NO_3_)_2_ to finally obtain a partially loaded Pb-**EMT**. The characterization has been primarily carried out by atomic-resolution transmission electron microscopy imaging that has been acquired by combining ADF and ABF detectors to obtain atomically resolved data of the framework and on the extra framework cations. These observations have been correlated with the data obtained by three dimensional electron diffraction tomography (3D-EDT). The existence of **FAU** intergrowths has also been analyzed based on this technique.

## 2 Methods

### 2.1 Synthetic Procedures

#### 2.1.1 Pb-EMT

**EMT** was synthesized using a previously reported method.^23,40^ Sodium aluminate (1.45 g) and a 50% NaOH solution (1.21 g) was added to 7.8 g of deionized water, together with 1.76 g of 18-crown-6 and stirred vigorously. Next, 15.4 g of a homogeneous silica solution (LUDOX AM-30) was added with stirring and incubated for 24 hours at room temperature to incubate. The mixture was placed in a 45 mL Parr pressure vessel and heated for 12 days at 110 °C. The product was filtered and washed with copious amounts of deionized water and then calcined in air at 540 °C for 16 h.

The potassium-ion exchange was performed as follows: 4 g of EMT was added to 500 mL of a 0.1 M KNO_3_ solution and stirred overnight. The powder was recovered and washed with 1 L of distilled water and air-dried overnight. The exchange procedure was repeated further two times.

The lead-ion exchange was carried out as follows: 2 g of EMT was added to 500 mL of a 0.1 M Pb(NO_3_)_2_ solution and stirred overnight. The powder was recovered and washed with 1 L of distilled water and air-dried overnight. The exchange and washing procedures were repeated further two times.

### 2.2 Characterization

#### 2.2.1 Scanning Electron Microscopy

STEM analyses were carried out on a JEOL Grand ARM 300, equipped with a cold field-emission gun (cold FEG) and operated at 300 kV. The column was fitted with a JEOL double spherical aberration corrector, which was aligned prior to the analyses to assure a maximum spatial resolution of 0.7 Å in the scanning mode. The microscope was also equipped with a JEOL EDS and a Gatan Quantum Energy Filter for spectroscopic measurements.

#### 2.2.2 3D-EDT

The 3D-EDT data were collected using JEOL.Shell software in a JEOL JEM2100 Plus electron microscope operated at 200 kV in the transmission electron microscope (TEM) mode. A total collecting angle of 120° was obtained by tilting the sample holder from −60 to +60°. The intensities were extracted with EDT.Process software. Finally, the structure was solved with Sir2014.^41^

#### 2.2.3 Energy-Dispersive X-ray Spectroscopy (EDS)

EDS data were collected using a JEOL JEM2100 Plus operated in the STEM mode with an accelerating voltage of 200 kV.

#### 2.2.4 Scanning Electron Microscopy (SEM)

The scanning electron microscopy (SEM) images were collected on a JEOL JEM7800 Prime with a work distance of 7 mm under a landing voltage of 1 kV.

#### 2.2.5 STEM Simulation

The simulated ADF-STEM and ABF-STEM images along [100] were obtained with software QSTEM.^42^ A supercell of 150 × 150 × 150 Å^3^ was constructed for pure silica EMT. The probe array was 600 × 600 pixels with 0.032 Å resolution and a slice thickness of 1 Å. The detector geometry inner and outer angles were 50–200 mrad for ADF and 5–40 mrad for ABF.

#### 2.2.6 Image Treatment

To minimize the beam damage, the electron dose was significantly reduced in comparison with standard STEM conditions. For the current experiments, the total time for image acquisition ranged from 6 to 10 s, 1024 × 1024 pixels using an electron dose of 1120–2800 e^–^/Å^2^. Under these conditions, images could be directly interpretated and all intensity analyses were performed over raw data. However, for a clearer visualization of the frameworks and of the light cations, the Random Noise 2D difference filter was used^43^ from HREM Research Inc.

## 3 Results and Discussion

**Figure 1:**
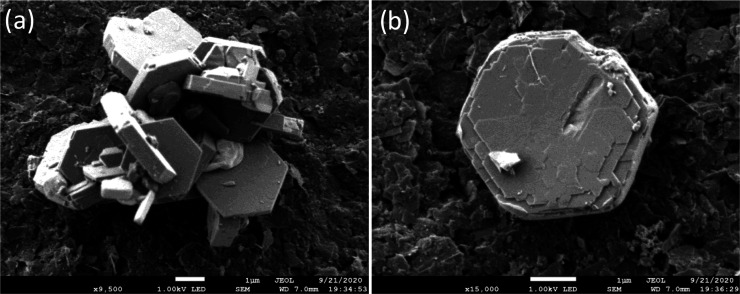
(a) Low-magnification SEM image of several **EMT** particles. (b) SEM image of an isolated **EMT** crystal with a hexagonal plate morphology.

The **EMT** framework type is a zeolite belonging to the hexagonal crystal family and **P**6_3_/**mmc** space group. The lattice constants previously reported are *a* = *b* = 17.215 Å, *c* = 28.082 Å, α = β = 90°, and γ = 120°. The morphology of the obtained **EMT** particles was in agreement with the hexagonal symmetry presenting well-defined hexagonal particles with a thickness up to 500 nm and widths that reached up to several microns, see Figure 1.

This framework is formed by double six rings (d6Rs) and sodalite (*sod*) cages that when connected, form faujasite layers (layer unit), with 12-membered rings formed along the *b* and *c* directions. The stacking sequence of these layers allows the formation of either **EMT** or **FAU**. By AA′ stacking of faujasite sheets, **EMT** is obtained, where the adjacent two layers are related to each other through a mirror plane. Alternatively, in **FAU**, the stacking sequence is ABC, where the connectivity exhibits inversion symmetry between successive layers.

For **EMT**, along the *b-*axis, the 12MRs run parallel to the observation axis while the layer unit runs perpendicular along the *c-*axis in an AA′ stacking. The chemical formula for calcined EMC-2 has been reported to be [Na_20_(H_2_O)_6_][Si_76_Al_20_O_192_]-**EMT**, where the 20 Na^+^ cations that compensate the presence of Al tetrahedra sit in three crystallographic sites at X_2_: 0.5890, 0.1770, and 0.0470; X_3_: 0.6667, 0.3333, and 0.6270; and X_4_: 0.3571, 0.1790, and 0.3880 with 0.25, 0.32, and 0.33 site occupancy, respectively; see Figure S1. In Figure S1, the T (T = Si and Al) atoms are presented as blue, brown, purple, and green spheres corresponding to the different crystallographic sites, O in red, while X_2_ appears in yellow, X_3_ in light blue, and X_4_ in pink, all of them associated to the 6SRs of the *sod* cages.

### 3.1 Analysis of FAU Intergrowths

**Figure 2:**
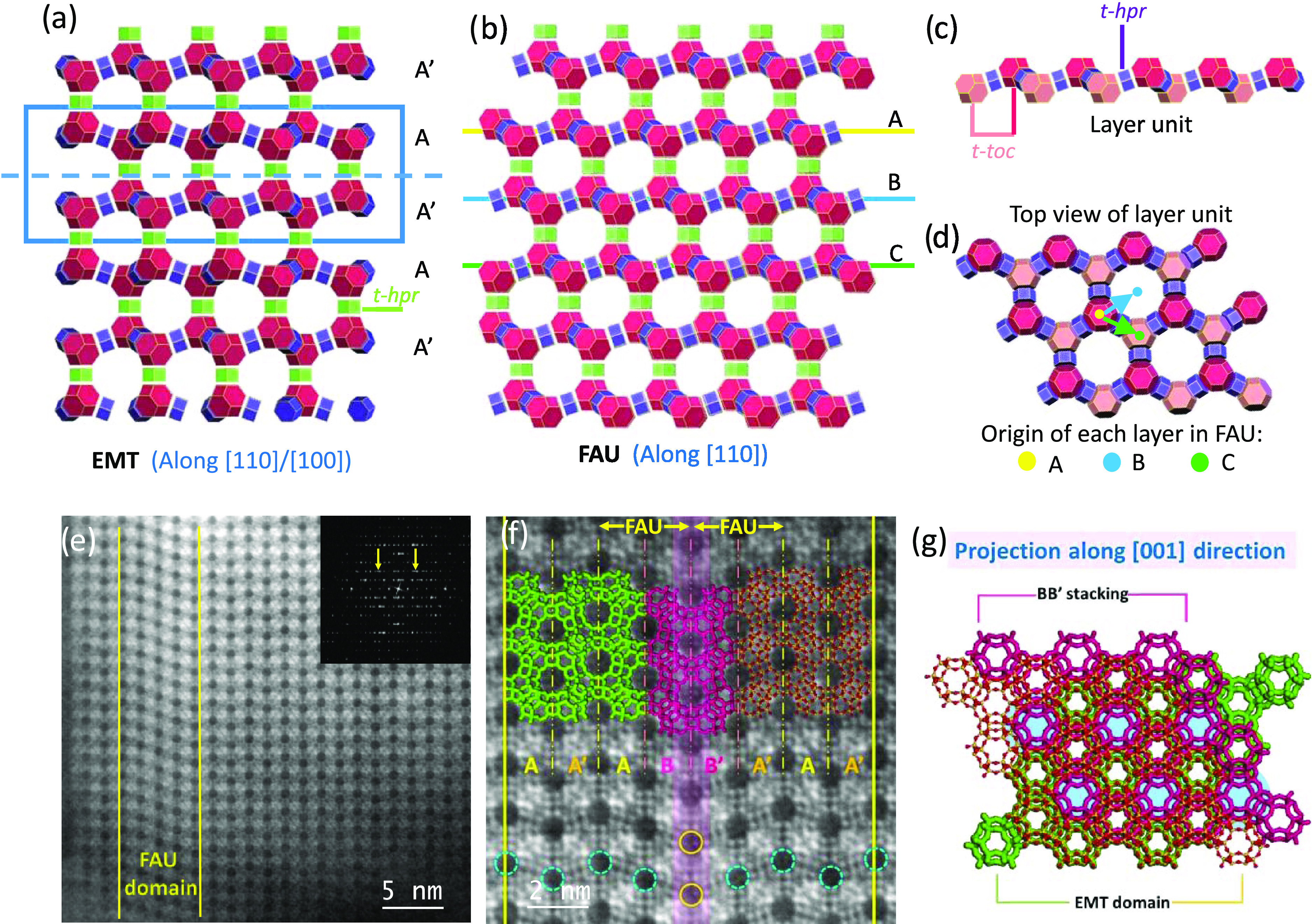
(a) **EMT** model along the [110]/[100]_EMT_ orientation. (b) **FAU** model along [110]. (c) Schematic model of a layer unit (faujasite layer), front view. (d) Top view of the model corresponding to a layer unit. The origins for the subsequent layers are marked by yellow, blue, and green dots. Color code: rose cages correspond to *t-toc* (sodalite cages); *t-hpr* (double six-membered ring, D6R) appear in green (between layers) and in purple (within per layer) cages. (e) C_s_-corrected STEM-ADF micrograph recorded along [110]/[100]_EMT_ with an **FAU** intergrowth marked by yellow lines. The Fourier diffractogram (FD) is shown in the inset with yellow arrows pointing at the diffuse streaks. (f) Closer observation of another crystal where two **FAU** domains are jointed by a mirror plane BB′. The dashed blue circles correspond to the 12MRs, while the yellow ones denote the partially blocked 12MRs within the twin plane. (g) Schematic representation of the structure model of (f) along the [001]_EMT_ direction.

It has been previously mentioned that due to the structural similarities (the use of the same building units differing only on how the layers stack), **FAU** and **EMT** can coexist in the same crystals.^23,37,44^ Along the [110] orientation, both hexagonal **EMT** and cubic **FAU** exhibit different ways of pillaring the faujasite layers, where for **EMT**, there is an AA′AA′... stacking (Figure 2a) and for **FAU** (Figure 2b), it follows ABC... pillaring. These layers are formed by sodalite cages (*t-toc*), in pink, linked by d6Rs (*t-hpr*), in purple, which are subsequently also connected by d6Rs (*t-hpr*) shown in green between different layers. An isolated faujasite layer unit is depicted in Figure 2c.

Because of these structural similarities, it could be expected that intergrowths of both phases due to the layer stacking sequence in a defective way would exist. In fact, there are many cases where **EMT** has been crystallized in the presence of **FAU**.^45−47^ For **EMT**, A and A′ layers are mirror related to each other.^48^ While in **FAU**, layer B is shifted (1/2a, 1/2b, 1/2c) relative to layer A, and layer C is shifted (1/2a, 0, 1/2c) relative to layer A. The origin of the A, B, and C layers is marked in Figure 2d. Large *t-wou* and *t-wof* cages for **EMT** and *t-fau* cages for **FAU** are formed between layers. *t-wof, t-wou*, and *t-fau* have three, five, and four 12MRs windows, respectively.

Figure 2e corresponds to the C_s_-corrected STEM ADF image of an **EMT** crystal with structural defects in the framework evidenced by the diffuse spots (yellow arrows) in the Fourier diffractogram (see inset). This region, of approximately 6 nm along the [110]/[100]_EMT_ zone axis, corresponds to a **FAU** domain enclosed by **EMT** regions. In another crystal, the sequence AA′ABB′A′AA′ can be observed, as shown in Figure 2f. This region is formed by two **FAU** domains connected through a mirror plane (defined by BB′ layers) enclosed within the **EMT**. For direct interpretation, the schematic model has been superimposed displayed in different colors to distinguish the three zones, as shown in Figure 2f. The left (green) and right (yellow/red) regions, corresponding to the **EMT** framework, allow the clear visualization of the 12MRs, marked by blue dashed circles. While in between, the twin plane is marked in pink color. By observing this structure along the [001] projection, as shown in Figure 2g, these two **EMT** domains would overlap, where the 12MRs marked with a blue background would be blocked by sodalite cages from the twin plane, BB′, as shown in Figure 2f. The large 12MR windows of the BB′ stacking would also be blocked by the **EMT** sodalite cages from both sides, as shown in Figure 2g. By observing Figure 2f (yellow circles), the existence of a faint signal inside the 12MRs in the twin plane that is associated with the presence of sodalite cages from the **EMT** region can be appreciated, partially blocking these channels. These 12MRs are fully empty in the **EMT** area (blue dashed circles).

### 3.2 Analysis of the EMT Framework and Extra Framework Cations

It is possible to synthesize the **EMT** framework with different Si/Al ratios, but in general terms, the most common zeolite obtained has been EMC-2 with the chemical formula |Na_20_(H_2_O)_6_| [Si_76_Al_20_O_192_].^23,24^ In this work, energy-dispersive X-ray spectroscopy (EDS) analyses were used to estimate the chemical composition of the current **EMT** (Table S1). The results obtained were reasonable and in agreement with the data reported. The most relevant differences with previous studies were that K^+^ was also used as a counter ion together with Na^+^, and that Pb^2+^ was also detected due to the ion exchange. Based on these results, the electroneutrality would be maintained as the sum of the positive charges from the cationic species would be 18 that would compensate 20 (AlO_4_^–^) of the framework. The small mismatch between the cations and aluminum is related to a slight experimental error of the technique or to the presence of protonic species H^+^ from water. In fact, the excess of O detected could be associated with water adsorbed in the pores. For simplicity, hereafter, we will refer only K^+^ when considering both Na^+^ and K^+^, as due to their low content and a similar atomic number, it is not possible to distinguish between both metals.

Annular dark-field (ADF) together with annular bright-field (ABF) imaging modes can reveal unique information, especially at a local level. Using an ADF detector, the electrons scattered at a high angle are used to form the images, allowing the visualization of heavier elements as the signal is proportional to *Z*^1.6–2^,^49,50^ where *Z* is the atomic number. On the other hand, ABF provides complementary information of the framework, as it is more sensitive to light compounds, even with a higher spatial resolution.^36,38,39^

**Figure 3:**
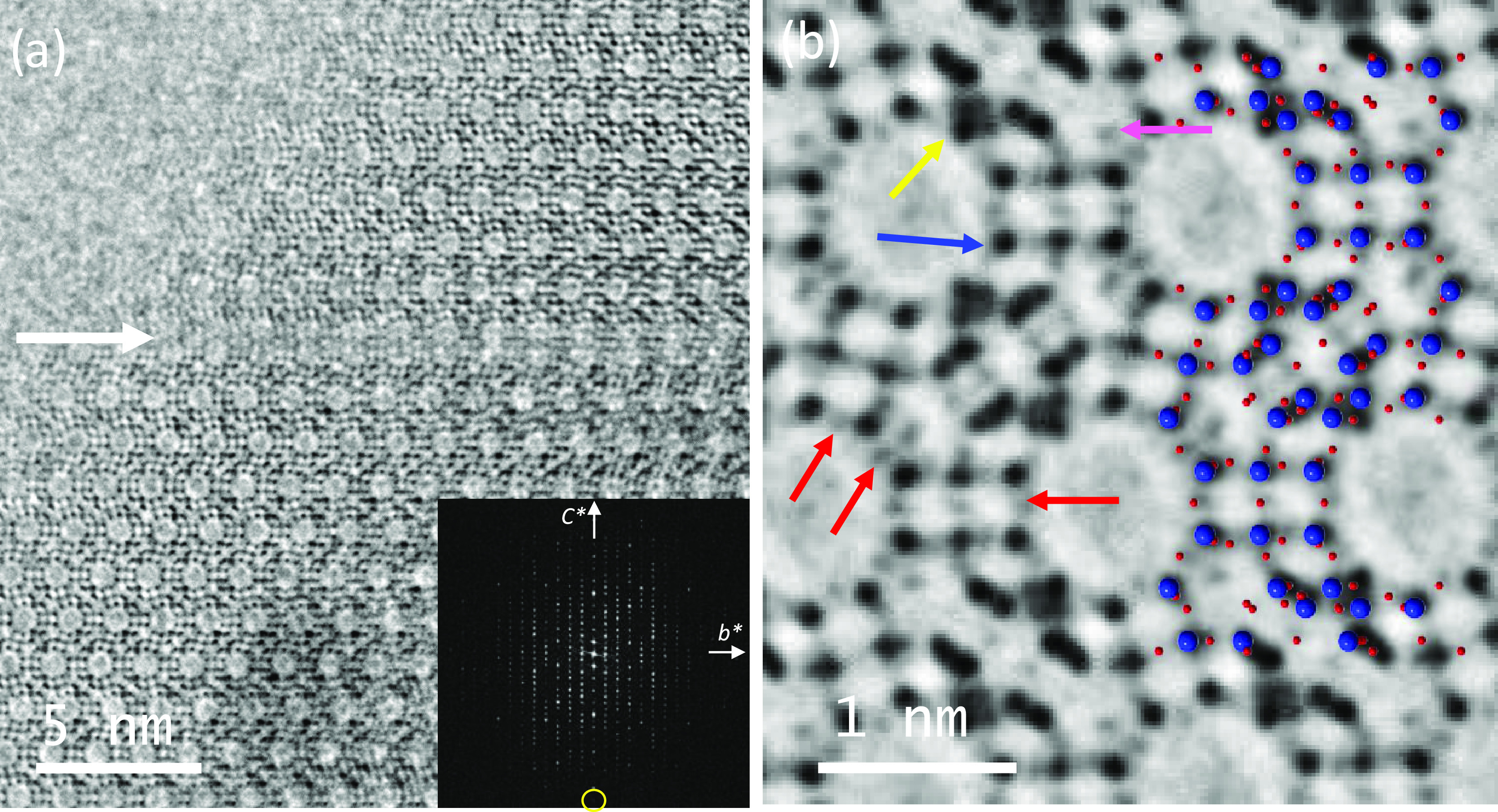
C_s_-corrected STEM-ABF data of Pb-**EMT**. (a) Atomic-resolution data along the [100] projection with the FD shown in the inset. The white arrow corresponds to a structural defect. The yellow circle in the FD indicates the maximum transfer information corresponding to the 00022 spot. (b) Symmetry *p*2*gm* averaged data with the framework model superimposed. The red arrows point at O bridges, the blue one corresponds to the T atoms, and the yellow and pink ones to extra framework signals.

**Figure 4:**
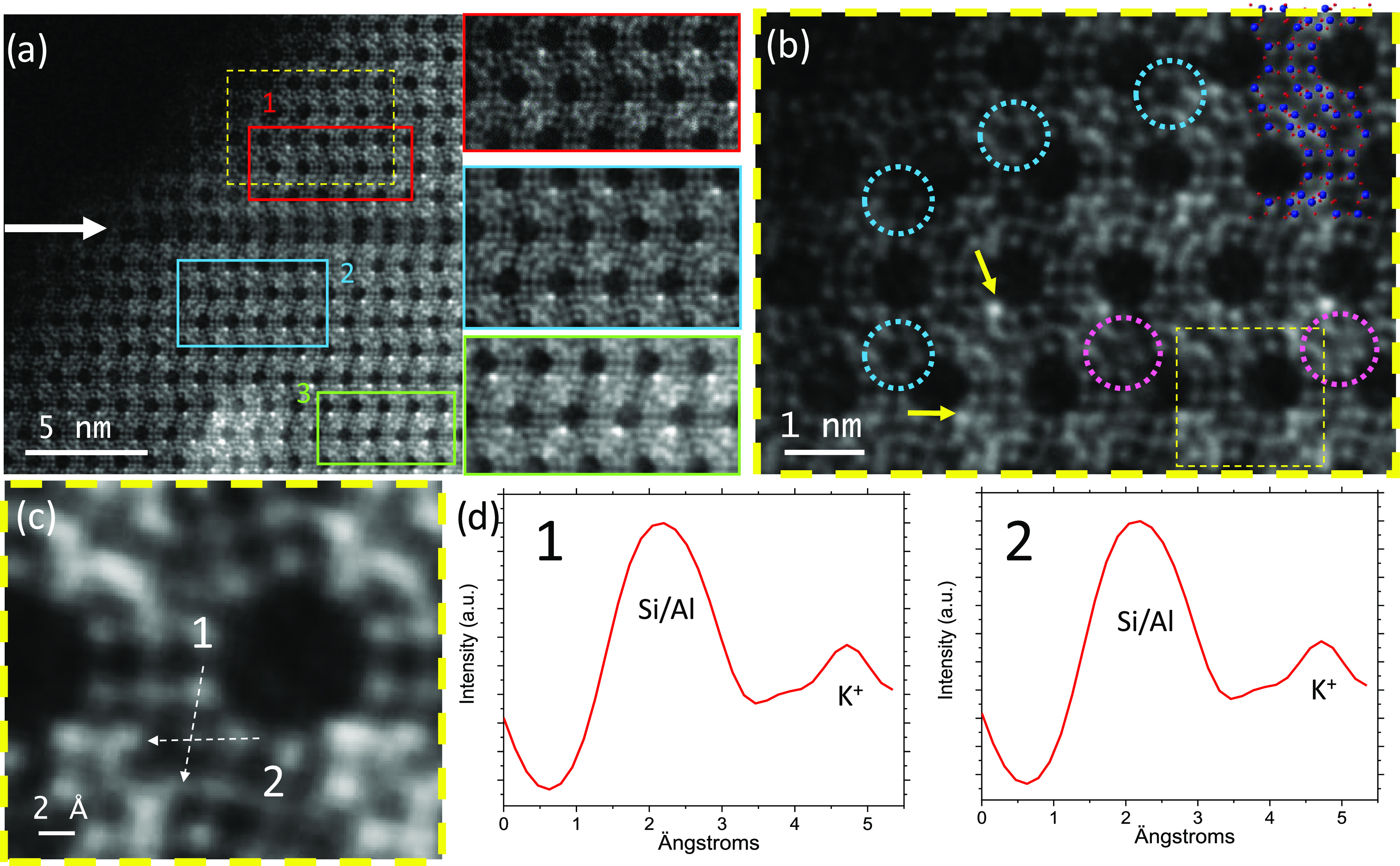
(a) C_s_-corrected STEM-ADF along [100] of Pb-**EMT**. The three colored rectangles correspond to the areas where the intensity analysis was performed. The dashed yellow rectangle indicates from where figure (b) was obtained from. (b) Closer observation of the framework where the bright spots are marked by yellow arrows. An empty s6R is denoted by a dashed blue circle. The dashed pink circle corresponds to s6R with a cation inside. (c) Enlarged image of s6R where the two arrows indicate where the intensity profiles were extracted from, numbered as 1 and 2. (d) Intensity profiles along 1 and 2.

For the **EMT** framework type, the most suitable projections for the structural observation are [110]/[100] two equivalent orientations with the minimum atomic column overlapping; hereafter, we will refer only to [100] for simplicity. Figure 3a corresponds to the C_s_-corrected STEM-ABF micrograph of Pb-loaded **EMT** zeolite along the [100] orientation; in this case, the atomic columns appear as black dots and the big pores, which are clearly observable corresponding to 12MRs. The white arrows point to a structural defect resulting in the merging of two faujasite sheets, which breaks the AA′A stacking; the FD shown in the inset proves that the spatial resolution that can be achieved is at least 1.2 Å represented by the 00022 diffraction spot, marked by a yellow circle. The symmetry-averaged image is depicted in Figure 3b; although the ABF data already present a sufficient degree of quality to analyze the structure at an atomic level, the averaged data are displayed to facilitate the observation of some particular aspects. Symmetry averaging image treatment presents certain advantages as it allows discussion about plane symmetry elements and it significantly increases the signal-to-noise ratio. On the other hand, it introduces undesired periodicity, which is disadvantageous for nonperiodic materials such as zeolites with uneven cationic distribution. Here, symmetry averaging has been used to improve the signal-to-noise ratio; for **EMT** topology, the reported space group is **P**6_3_/**mmc** (194); therefore, its plane group along [100] should be *p*2*gm*. The averaged data assuming this space group is depicted in Figure 3b, highlighting (i) T atoms (blue arrow) and (ii) oxygen bridges (red arrows) and additional signals that are not part of the framework (pink and yellow arrows). To facilitate the interpretation, the model of the framework has been superimposed where Si appears in blue and O in red; for clarity, the presence of extra framework cationic species has been omitted. Figure S2 compares the experimental data (Figure S2a) with the simulated image (Figure S2b) of the all-silica framework (without cationic species to highlight the differences between the bare framework and the experimental data); in both cases, colored arrows have been used to point the framework atoms and the extra framework sites. Figure 3b exhibits excellent image quality and it corroborates the feasibility of ABF imaging for the observation of light compounds as oxygen bridges. In addition, the two distinct signals that do not belong to the framework can be inferred to be cationic species; however, an aspect that needs to be considered is that, to obtain this image, symmetry restraints have been introduced and the presence of those extra framework species does not necessarily need to be periodic. To evaluate this aspect and to study the possible introduction of Pb^2+^ in the pores after ion exchange, we turned to C_s_-corrected STEM-ADF without imposing any symmetry averaging, as shown in Figure 4. Figure 4a depicts the corresponding C_s_-corrected STEM-ADF data of the Pb-**EMT** zeolite on the [100] projection; here, the contrast is reversed with respect to the ABF data, where the atomic columns appear as bright spots and in which the 12MRs are observed together with the d6Rs and the sodalite cages. Interestingly, a strong contrast is observed as bright spots, which appear at specific sites in a nonperiodic manner. The areas marked by rectangles with different colors, numbered 1–3, were enlarged to analyze the contrast variations at different regions of the crystal, while the additional dashed yellow rectangle corresponds to the area that is magnified, as shown in Figure 4b. The intensity profiles along several sodalite cages were extracted (Figure S3), observing some maxima corresponding to the bright spots. In Figure 4a, the 12MRs are clearly visualized as empty pores; meanwhile, the bright spots would be placed at the sites where Na^+^ has been proposed to occupy, corresponding to the X_3_ and X_4_ sites. This observation is in agreement with the data obtained with an ABF detector (see Figure 3b, yellow arrow), which indicates a very strong signal (that appears periodically due to the symmetry-averaging) near the oxygen bridges. Such signal observed in the ABF and in the ADF cannot derive from the O bridges due to their low scattering factor, and therefore, it is owed to the presence of single Pb^2+^ sites (single atoms, or atomic columns). Due to the low initial occupancy of the extra framework cations (K^+^ and Na^+^ here), it cannot be estimated whether Pb^2+^ is in the form of single atoms or if there are several Pb^2+^ on the same column. Nevertheless, X_3_ and X_4_ sites correspond to two distinct atomic sites with different coordinates; however, due to their proximity in the projected image ≈0.5 Å, it cannot be initially distinguished whether Pb^2+^ replaced X_3_, X_4_, or both.

For in-depth investigation of the ion-exchange capability and therefore the location of the cations, the schematic model of a sodalite cage is shown in Figure S4a. The color code is same as in Figure S1, which displays T_3_ (T = Si, Al) crystallographic sites in purple, forming s6R, where the light-blue sphere corresponds to the cationic site at X_3_. Blue T atoms correspond to T_1_, brown spheres are the T_2_ sites, purple T_3_, and green T_4_. Cations at X_4_ are marked in pink, while cationic sites at X_2_ appear in yellow. If Pb^2+^ would occupy the X_4_ site, this would correspond to the location of Pb^2+^ on top of the S6Rs of the *t-toc* cage; see Figure S4a. On the other hand, if Pb^2+^ would replace X_3_, this one would be in a similar position, on top of another S6Rs of the *t-toc* cage but closer to the framework. Figure S4b presents an entire unit cell with the same color code, along this projection, the proximity between X_3_ and X_4_. It is important to mention that no experimental evidence was found of Pb^2+^ occupying the X_2_ sites, which in the projected image would be inside s6Rs very close to T_1_ atoms.

The other extra framework signals which were not as intense as Pb^2+^ were observed within s6Rs. In this case, the signal marked by a pink arrow in the ABF data (Figure 3b) and by a pink dashed circle in Figure 4b, would correspond to K^+^ cations (based on the chemical analyses, the K^+^ content is much higher than Na^+^, although because of their similar atomic numbers, it would not be possible to distinguish between them). These extra framework species were not periodically distributed along the entire framework, as empty s6Rs could also be identified; see dashed blue circles in Figure 4b. This observation could be associated with the presence of aluminum in the framework, suggesting that the aluminum was not periodically distributed as the empty s6Rs would be linked to the absence of Al at those sites; meanwhile, the extra framework cationic presence suggests the existence of Al around them. Figure 4c depicts an enlarged image of 12MR, obtained from the yellow dashed rectangle in Figure 4b, where K^+^ can be located within s6R. Two different intensity profiles were plotted in Figure 4d, along the arrows 1 and 2, where two peaks are present: the first one and more intense corresponds to an atomic column of the framework, Si or Si + Al, while the other belongs to K^+^. The measured projected distances were *d*_1_ = 2.05 Å, corresponding to the distance between a T_2_ cation and *d*_2_ = 2.28 Å, corresponding to the distance between the T_4_ cation; these measurements (slightly shorter) are in agreement with the data reported for Na-**EMT** (for X_4_ sites), where projected distances are 2.20 and 2.42 Å, respectively. Furthermore, no evidence for the presence of K^+^ or Pb^2+^ at X_2_ sites was found in any of the equivalent sites.

Based on these observations, Pb^2+^ would preferentially replace the cations at the X_3_ sites, as shown in Figure 4b (yellow arrows). Despite that the X_3_ and X_4_ sites are too close to be distinguished, we have not observed evidence for the presence of Pb^2+^ at s6Rs, denoted by pink dashed circles in Figure 4b, which would correspond to K^+^ at X_4_ sites. Therefore, as K^+^ at X_4_ sites has two equivalent positions if Pb^2+^ would enter at X_4_, this stronger signal would be observed at the sites indicated by a pink circle and at the sites pointed by yellow arrows. As this is not the case, it is possible to conclude that Pb^2+^ would only go into the sites denoted as X_3_; in fact, as K^+^ is at X_4_, the total signal detected at X_3_ would be mostly owing to Pb^2+^ but with a contribution from K^+^ present at X_4_. Despite that, the Pb^2+^ signal was not homogeneously observed along the entire crystal; it always occupied a particular crystallographic site. However, due to the low occupancy of the extra framework cations, 0.33 for X_4_, it does not appear along the entire crystal. From these observations, it can also be inferred that Pb^2+^ would compensate the electric charge of the cations at X_2_ sites; as seen from the experimental data, no signal was detected at these positions, suggesting that the cations were removed from these sites but Pb^2+^ did not fill the positions; the electroneutrality was maintained as one Pb^2+^ would compensate two monoatomic species of either Na^+^ or K^+^. For comparison, simulated data were compared with the experimental data; see Figure S5. Figure S5a corresponds to the simulated image of the **EMT** framework with no cations added to the structure. Figure S5b displays the same image with different amounts of K^+^ and Pb^2+^ added to the system. The blue dashed circle corresponds to s6R where K^+^ is not observed; the number of K^+^ that was placed at that site was six atoms in that column; therefore, for a given thickness of 8 nm (≈4 unit cells), six atoms of K^+^ per column were not detectable. The pink dashed circle corresponds to K^+^ at s6Rs (18 atoms per column, which are clearly visible). Meanwhile, the yellow arrow points at the signal owing to Pb^2+^ (in this case, the column was filled by six atoms). Figure S5c corresponds to the experimental data obtained. To ignore that the strong signal observed can be the result of the possible interaction of the framework atoms with the cations (due to their proximity on the projection image), additional simulations with different cationic contents are depicted in Figure S5d,e.

### 3.3 Framework Analysis and Crystallographic Determination of Pb^2+^ and K^+^ Positions

**Figure 5:**
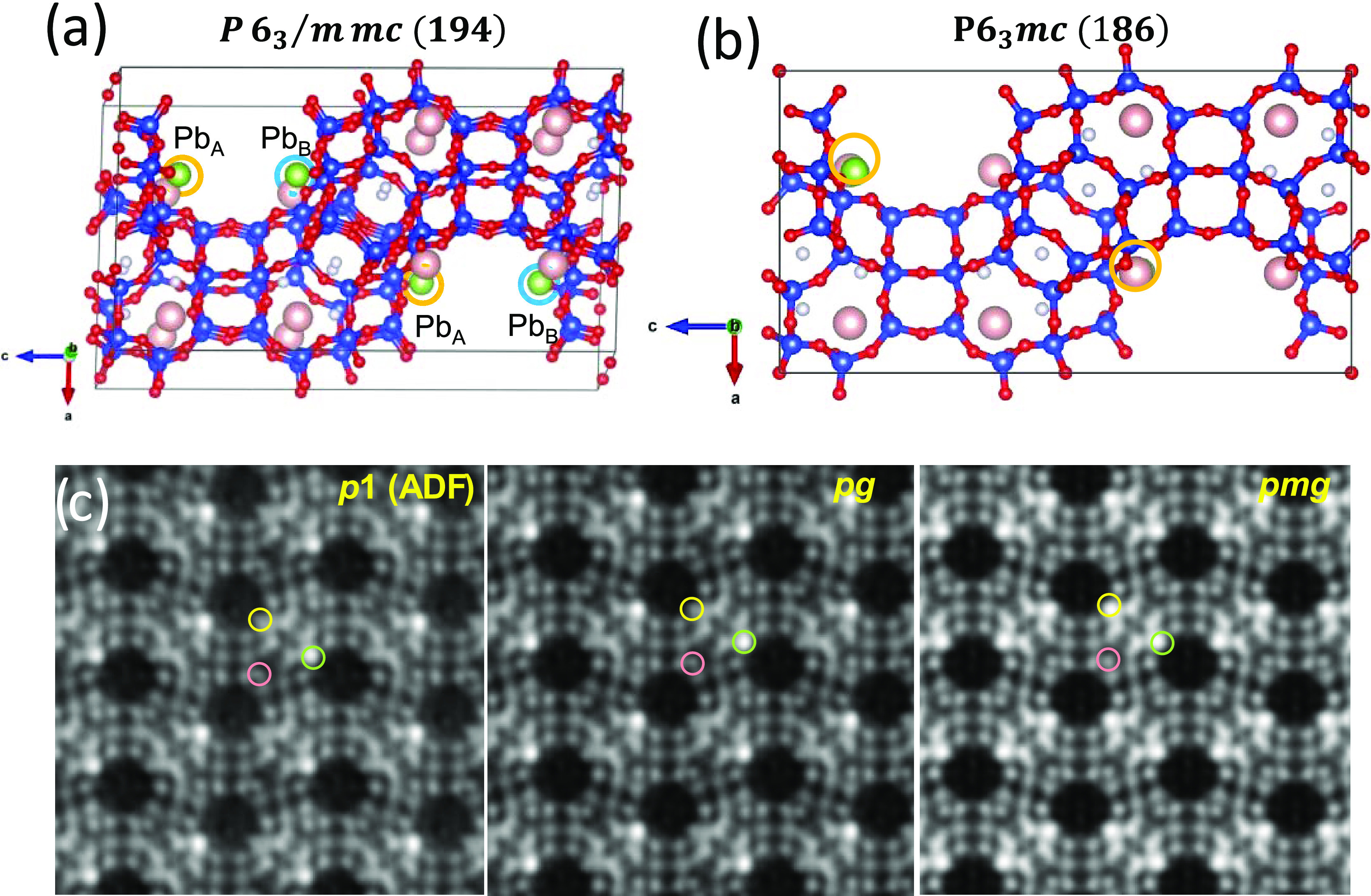
(a) Structural solution using **P**6_3_/**mmc** (194). (b) Structural model after removing Pb_A_ or Pb_B_, leading to a lower symmetry **P**6_3_**mc**. (c) C_s_-corrected STEM-ADF data after symmetry averaging. The colored circles indicate the same crystallographic sites in each image, green corresponds to Pb^2+^, rose corresponds to K^+,^ and yellow would be the equivalent site to Pb^2+^ marked by a green circle.

How image analysis at the atomic level can provide local information of the zeolitic framework and extra framework cations has been proved so far. On the other hand, electron diffraction can deliver more general information as the solution obtained is based on an average over several unit cells. The structure of Pb-**EMT** zeolite was solved based on the diffraction intensities extracted from the 3D-EDT using direct method. Figure S6 displays the extracted electron diffraction patterns along the main crystallographic orientations. The patterns shown in Figure S6a–c belong to c*, b*, and a* projections, respectively. Along the c*-axis, this zeolite exhibits 6-fold symmetry, while two perpendicular mirror planes are present along the other orientations, running along a*, b*, and c* axes (see Figure S6b,c). From the *hhk* plane (Figure S6d), the reflection condition 00*l*: *l* = 2*n* was confirmed, marked by a blue rectangle. For *hk*0, *hk*1, *hk*2, and *hk*3 planes, *hhl* and *h-hl* (*l* = 1, 2, 3, 4) reflections are marked with green and pink rectangles, respectively. Reflections *h-hl* appear in all of the four layers, while *hhl* only appears when *l* = 2*n* (Figure S6e–h). These reflection conditions are in agreement with the extinction symbol *P*_ _ *c*. Thus, only three space groups fulfill these conditions **P**6_3_**mc** (186), *P*-62*c* (190), and **P**6_3_/**mmc** (194). These three were used to solve the structure, obtaining reliable results when **P**6_3_**mc** and **P**6_3_/**mmc** were used. The structure solution assuming the highest symmetry would be **P**6_3_/**mmc** (194) in agreement with the **EMT** topology,^24^ (Figure 5a). Based on the data extracted from 3D-EDT, (see Table S2 for atomic coordinates) Pb^2+^ cations would occupy the *t-wof* cage with 4 equiv sites, represented as green spheres in Figure 5a. Meanwhile, the light cations Na^+^/K^+^ situated within the large *t-wou* cages have 12 equiv sites per cell (rose spheres). These results are in concordance with the experimental atomic-resolution data presented in Figures 3 and 4 along the [100] projection (yellow arrows in Figures 3 and 4). Although the averaged solution from 3D-EDT suggested four equivalent positions, the experimental C_s_-corrected data revealed that Pb^2+^ did not occupy all of the four sites. Instead, Pb^2+^ would not fill the sites situated opposite to each other, corresponding to either Pb_A_ or Pb_B_ in Figure 5a. Based on these observations, if two Pb^2+^ atoms from either Pb_A_ or Pb_B_ sites are removed, the space group would lower its symmetry changing from **P**6_3_/**mmc** to **P**6_3_**mc** (see Figure 5b), where the two Pb^2+^ atoms (green spheres) are marked by yellow circles. On the other hand, light cations Na^+^/K^+^ were found at s6Rs of the *t-wou* cages (rose spheres). At this crystallographic site, some of the Na^+^/K^+^ symmetric equivalent sites overlap with Pb^2+^ along [110]/[100] projections, as shown in Figure 5b, but they are in different cages.

To reach out to these findings, we turned out to image analyses (Figure 5c). Figure 5c displays the ADF symmetry-averaged data obtained after using the plane groups marked in Figure 5c; *p*1 is the experimental data with no symmetry operation imposed, and only translation. The other two images were obtained after using *pg* (plane group from **P**6_3_**mc**) and *p*2*gm* (plane group from **P**6_3_/**mmc**). To clearly highlight the differences between the three images, color circles were superimposed in the figure; green would correspond to the strong signals associated with Pb^2+^, rose circle indicates the light signals from K^+^, and the yellow circle indicates equivalent sites for Pb^2+^, which would overlap the symetrically related sites of K^+^ marked by rose circles in Figure 5c, as shown in Figure 5b. Although Pb^2+^ and K^+^, green and rose circles, respectively, are equal for the three cases the major difference is indicated by the yellow circle as a strong signal is observed for *p*2*gm* that is absent for *pg* and for *p*1 (experimental data). Based on this significant difference, it is possible to conclude that Pb^2+^ did not fill the sites situated opposite to each other occupying two of the four symmetrically related sites and that the correct plane group should be *pg*, and therefore, the correct space group considering this cationic distribution is **P**6_3_**mc**.

By comparing the experimental data acquired by C_s_-corrected imaging at an atomic level, it was possible to elucidate preferential sites for Pb^2+^ and K^+^ as well as evaluate the ion-exchange capability and the sites that are more suitable to be initially exchanged. The electron diffraction data allowed the characterization over an averaged volume of the structure, and the results obtained were in agreement with that obtained by C_s_-corrected STEM, allowing us to conclude that Pb^2+^ is located in the *t-wof* cage and K^+^ is located in the *t-wou* cage. By imaging, it was possible to acquire local information showing the absence of cations at positions that should have been occupied based on the electron diffraction should be occupied. This is because the results obtained by electron diffraction were averaged over a relatively large area (100 nm, limited by the diffraction aperture used). Therefore, the atomic coordinates occupied by Pb^2+^ and Na^+^/K^+^, which were not part of the zeolite framework, were the result of not single atoms but the preferential sites for cationic occupancy that on average tend to go to those sites. These results illustrate the importance of combining electron diffraction with atomic-resolution imaging to achieve a complete characterization of the structure of zeolites, especially with the intention of gaining information at a local level.

## 4 Conclusions

In conclusion, a combination of various electron microscopy techniques has been used to analyze the structure and the ion-exchange capability of **EMT** zeolite, where K^+^ and Pb^2+^ were both introduced in the pores. By using a multitechnique approach, the particular features of Pb-**EMT** have been revealed. SEM data allowed the visualization of the morphology, obtaining symmetry elements, which facilitated the structural solution.

C_s_-corrected STEM observations displayed data with enough quality to obtain atomic-resolution information, even showing the oxygen bridges as well as cations Pb^2+^ and K^+^ that were atomically distributed over the framework. The atomic coordinates were determined by 3D-EDT. Both Pb^2+^ and K^+^ were found to be outside the sodalite cages and above the s6Rs of these cages, while Pb^2+^ was in the *t-wof* cages, K^+^ was in *t-wou* cages. Overall, atomic-resolution images provided insights of the local structure of Pb-**EMT** gaining information on the ion-exchange capability and on the location and periodicity of the extra framework cations, which helped achieving a more accurate structural solution. By the combination of 3D-EDT with the image analysis, it was concluded that crystal symmetry had to be reduced from **P**6_3_/**mmc** to **P**6_3_ mc.

## Supporting Information Available

Experimental details, including chemicals, synthesis, and characterization techniques (PDF)
