# Supplementary material for: Direct Imaging and Location of Pb2+ and K+ in EMT Framework-Type Zeolite
Source: J Phys Chem C Nanomater Interfaces. 2021 Mar 15;125(11):6461–70. doi: 10.1021/acs.jpcc.1c00550 (PMC8482759; doi:10.1021/acs.jpcc.1c00550)
Supplement: Supplementary file 1 — jp1c00550_si_001.pdf [file jp1c00550_si_001.pdf]

Supporting Information

Direct Imaging and Location of Pb^{2+} and K^{+} in EMT Framework Type Zeolite

Yaping Zhang[b], Daniel Smith[d], Jennifer E. Readman[d,*] and Alvaro Mayoral[a,b,c,*]

[a] Instituto de Nanociencia y Materiales de Aragón (INMA), CSIC-Universidad de Zaragoza, Zaragoza 50009.

[b] Center for High-Resolution Electron Microscopy (ChEM), School of Physical Science and Technology (SPST), ShanghaiTech University, 393 Middle Huaxia Road, Pudong, Shanghai

[c] Advanced Microscopy Laboratory (LMA), University of Zaragoza, 50018 Zaragoza, Spain

[d] School of Physical Sciences and Computing, University of Central Lancashire, United Kingdom

Figure S1. Unit cell represenation of EMT. The “T” atoms that would correspond to Si or Al are represented as blue, brown, purple and green speheres (T1 to T4 sites respectively). The cations in the framework appear in yellow for X2 site, light blue for X3 and pink for X4 site.

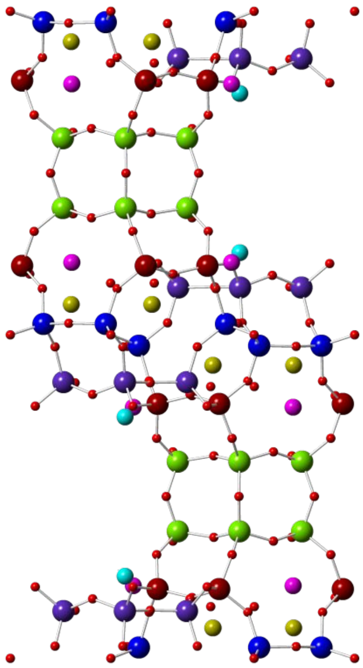

Figure S2. a) C_s-corrected STEM-ABF experimental symmetry averaged image of Pb-**EMT**. b) Simulated image of the framework, without cations. The arrows indicate: red O atoms, blue T atoms of the framworks, pink and yellow extraframework signals.

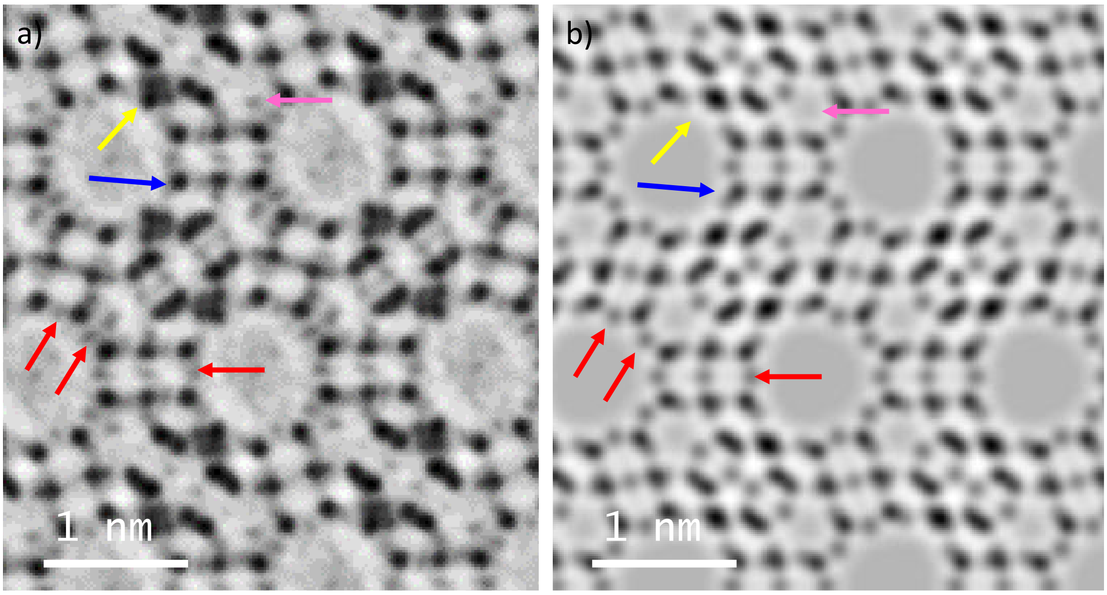

Figure S3. Three different intensity analyses perfomed along the white dashed reactangles. The most prominent signals are pointed by arrows on both the images and the correspondent intensity profiles.

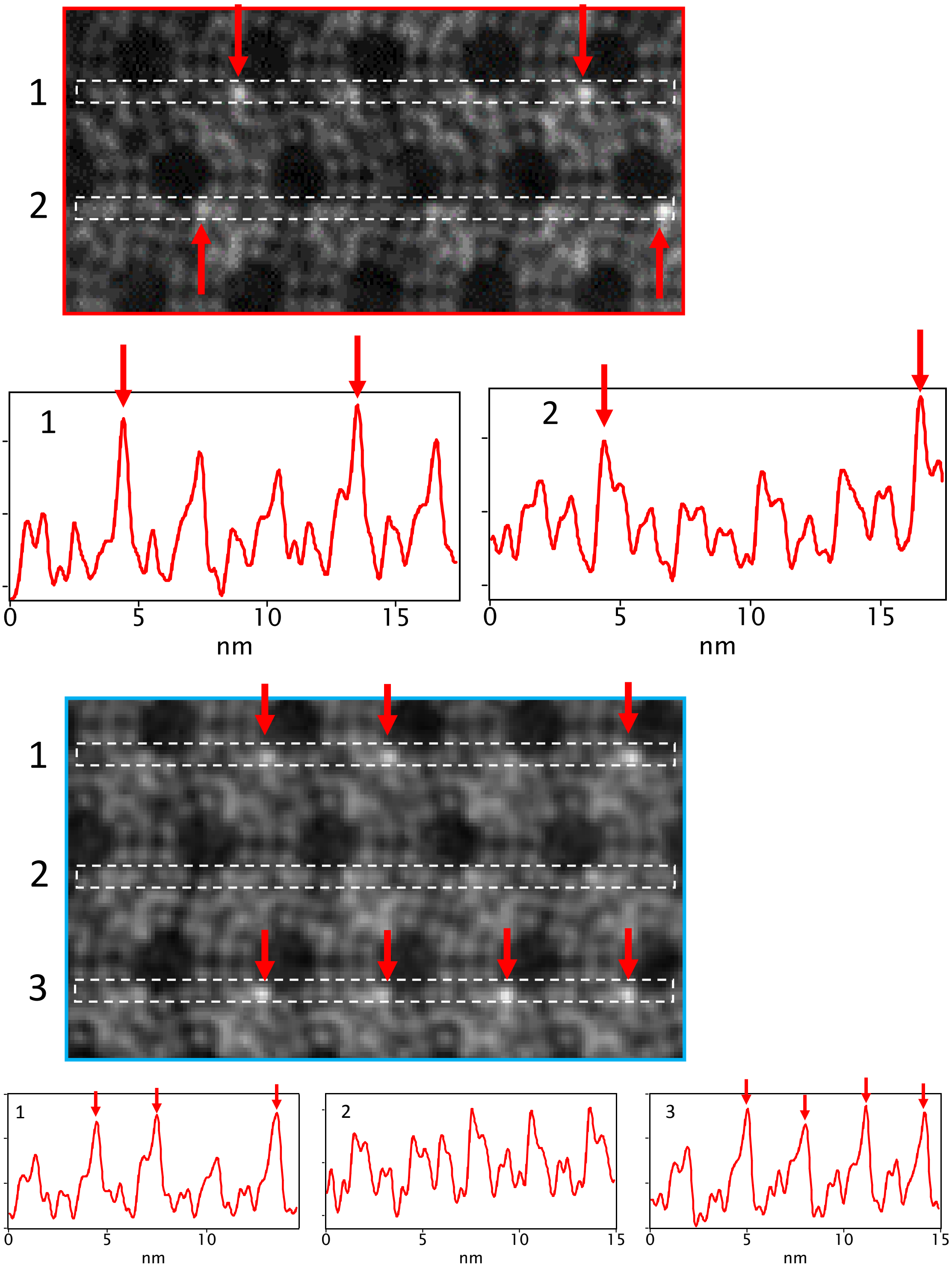

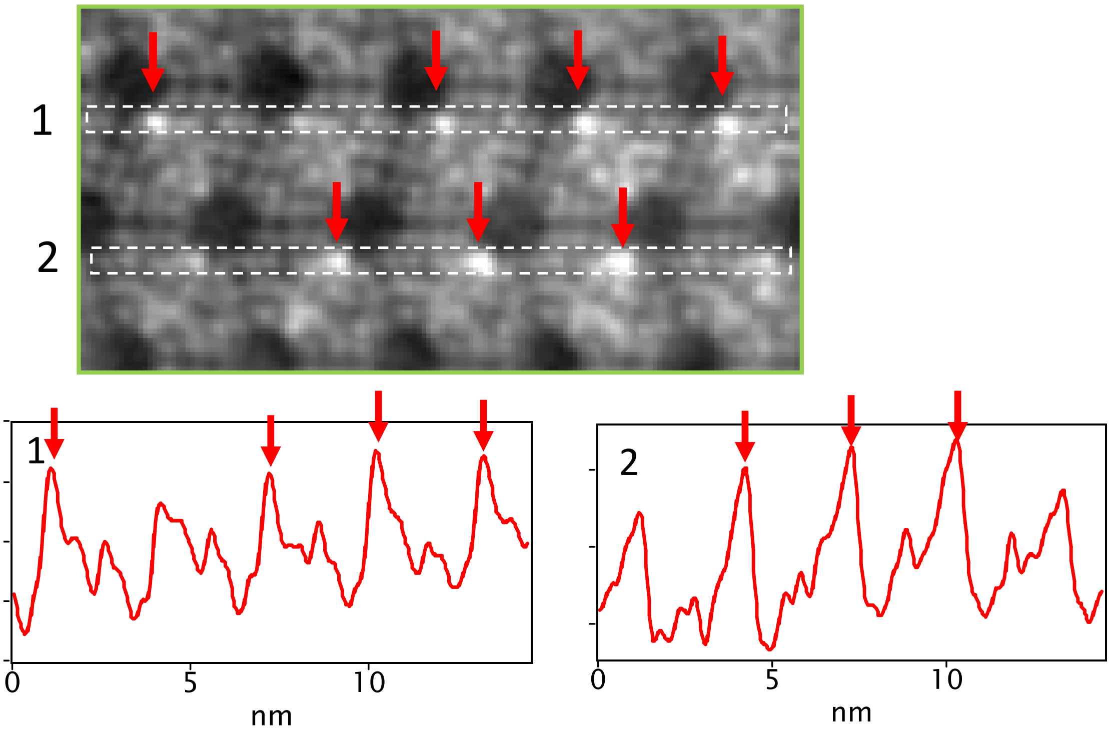
1
2
1
0
5
10
nm
2
0
5
10
nm

Figure S4. a) *Sodalite* cage with the cationic sites as yellow spehres for X2, blue X3 and pink spheres for X4. b) **EMT** unti cell using the same color code and along the same orientation.

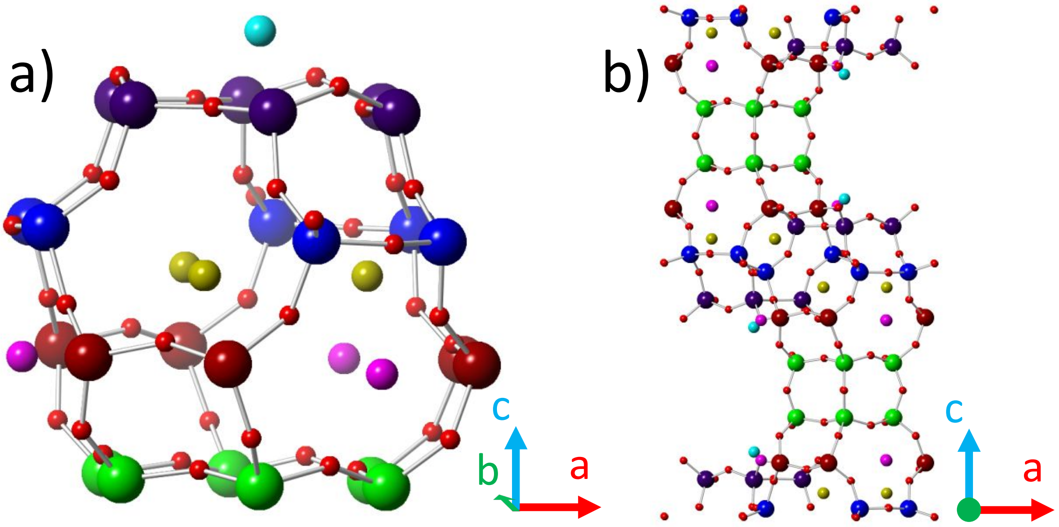

Figure S5. C_s-corrected STEM-ADF data of Pb-**EMT**. a) Simulated image of the framework with no cationic species. b) Simulated image of the framworks with different cationic content incorporated. Dashed blue circle corresponds to a s6R where K^+ is not visible. It correspons to 6 atoms in the column. The pink dashed circle indicates the presence of K^+ on the column, the K^+ content was 18 atoms. The yellow arrow points at the Pb^{2+} for 6 atoms in the column. The given thickness for the simulation was 8 nm. c) Esperimental image marking at the same cationic species. d) Simulated data comparing 2 atoms of Pb^{2+}, yellow arrow, versus 6 atoms of K^+. e) Simulated micrograph for a given thickenss of 124 nm with the amount of atoms per column (K^+ and Pb^{2+}) indicated in the figure.

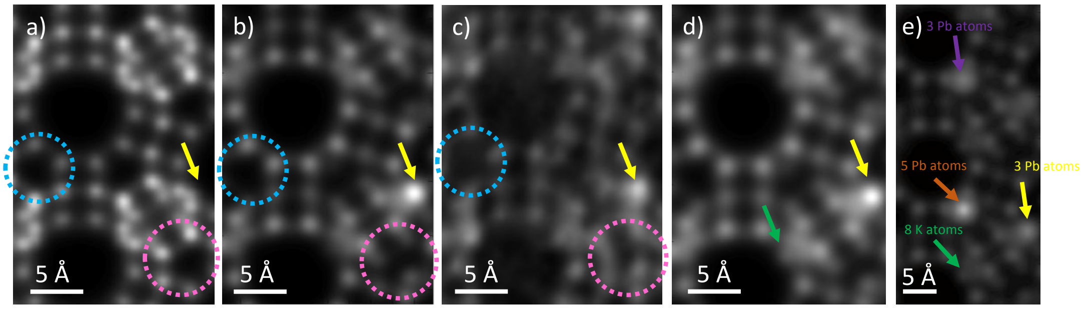

Figure S6. 3D-EDT slices along: a) c*; b) b*; c) a* and d) plane *hhk*. Blue box: *00l* reflections. It fits the reflection condition *000l*: *l*=2n; (e)~(h) Plane *hk0~hk3*. Green lines: *hhl* reflections; red lines: *h-hl* reflections. They confirmed reflection condition *hhl*: *l*=2n.

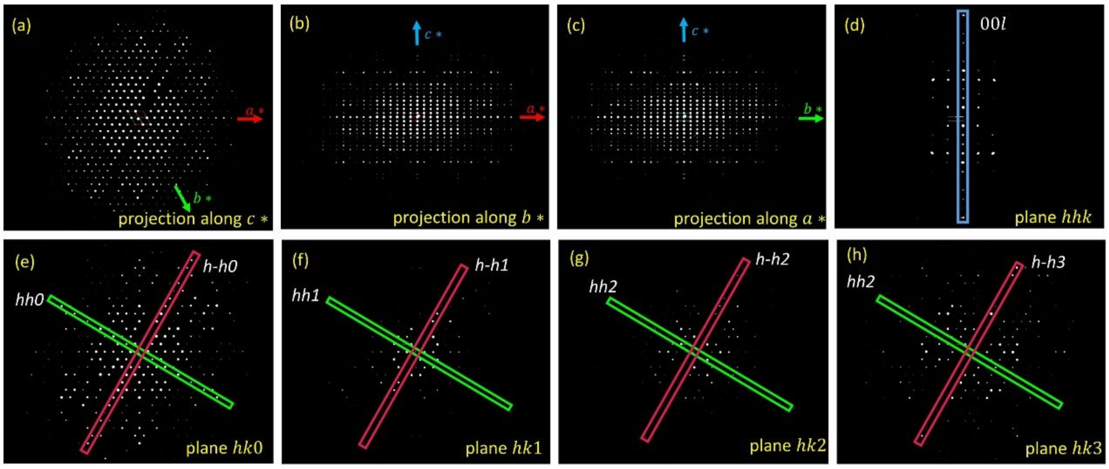

Table S1 Element analysis of low magnification STEM EDS mapping

Element	*O*	*Si*	*Al*	*K*	*Pb*	*I*	*Na*	*T(Si+Al)*
Average	205.0	75.9	20.1	10.2	2.2	0.2	3.9	96.0

Table S2 Atomic coordinates[i]

Symbol	*x*	*y*	*z*	*Occupancy*	*Site*	*Sym.*
T1	0.1535	0.4850	0.3042	1.0	24l	1
T2	0.1581	0.4877	0.5713	1.0	24l	1
T3	0.0360	0.4272	0.3900	1.0	24l	1
T4	0.0952	0.3670	0.4787	1.0	24l	1
O1	0.1296	0.4607	0.2500	1.0	12j	m..
O2	0.0703	0.4256	0.3393	1.0	24l	1
O3	0.1659	0.3317	0.4809	1.0	12k	.m.
O4	0.1331	0.4548	0.5195	1.0	24l	1
O5	0.0907	0.3979	0.4292	1.0	24l	1
O6	0.1850	0.5925	0.3108	1.0	12k	.m.
O7	-0.0688	0.3616	0.3952	1.0	24l	1
O8	0.2405	0.4809	0.5883	1.0	12k	.m.
O9	0.0000	0.2861	0.5000	1.0	12i	.2.
O10	0.0602	0.5301	0.4028	1.0	12k	.m.
O11	0.2378	0.4757	0.3189	1.0	12k	.m.
O12	0.1848	0.5924	0.5697	1.0	12k	.m.
K/Na	0.1647	0.3294	0.3768	1.0	12k	.m.
Na/H2Oa	0.2108	0.6054	0.4591	1.0	12k	.m.
Na/H2Ob	0.3333	0.6667	0.3575	1.0	4f	3m.

Symbol	*x*	*y*	*z*	*Occupancy*	*Site*	*Sym.*
Pb1a	0.3333	0.6667	0.6320	x[ii]	Group A[iii] 2	3m.
Pb2a	0.6667	0.3333	0.1320	x		
Pb1b	0.3333	0.6667	0.8680	1-x	Group B 2	
Pb2b	0.6667	0.3333	0.3680	1-x		

[i] The coordinates are given based on the $P6_3/mmc$.
[ii] When $x \neq 0.5$, the space group decreased to $P6_3mc$.
[iii] The Pb^{2+} cations tend to locate in two sites either in group A or group B in one unit cell.
